# DEVELOPING AND VALIDATING AN EXTRA-SHORT FORM OF THE LATER LIFE WORKPLACE INDEX (LLWI-XS) IN GERMANY AND THE US

**DOI:** 10.1093/geroni/igae098.2373

**Published:** 2024-12-31

**Authors:** Juergen Deller, Mattea Wehage, Julia S Finsel, Anne Woehrmann

**Affiliations:** Leuphana University of Lueneburg, Lueneburg, Niedersachsen, Germany; Leuphana University of Lueneburg, Lueneburg, Niedersachsen, Germany; Leuphana Uiversity of Lueneburg, Lueneburg, Niedersachsen, Germany; Federal Institute for Occupational Safety and Health (BAuA), Dortmund, Nordrhein-Westfalen, Germany

## Abstract

Employment of older individuals becomes more important. To identify organizational practices that foster the motivation, health, and performance of older employees, an efficient holistic assessment of relevant organizational factors is needed. The 80-item Later Life Workplace Index (LLWI) as well as the 29-item Later Life Workplace Index Short Form (LLWI-SF) provide such a measure. However, both measures are too long to be applied in large scale surveys, e.g., Health and Retirement Survey (HRS). Therefore, this paper describes the development and validation of the extra short form LLWI-XS, a 9-item measure covering all nine domains in order to achieve a highly efficient instrument to measure relevant organizational aspects. For each domain, one LLWI-XS item was developed deductively based on the domain description, indicators, and items from the original and short forms. This iterative process was done by an expert focus group who followed general item writing recommendations and standard practices to develop short and precise items applicable for large-scale surveys. The overall construct’s model fit and each domain’s correlations with criterion and discriminant validity measures are reported. The LLWI-XS items were developed and validated both in German and in U.S. American English. We provide researchers and practitioners with a validated extra-short measure to efficiently assess all nine organizational domains relevant for older employees to gain a comprehensive understanding of all critical areas of organizational influences on later life work. The scale is well suited to enable a screening of organizational practices for older workers in a population-based research context.

